# Assessing maternal satisfaction after caesarean section in government hospitals in Durban, KwaZulu-Natal: A qualitative study

**DOI:** 10.4102/safp.v68i1.6305

**Published:** 2026-06-24

**Authors:** Nokuthula Mbele, Zanine Moyce, David Bishop

**Affiliations:** 1 Discipline of Anaesthesiology, Pain Management and Critical Care, College of Health Sciences, University of KwaZulu-Natal, Durban, South Africa

**Keywords:** maternal satisfaction, caesarean section, communication, autonomy, pain management, breastfeeding support, Durban, South Africa

## Abstract

**Background:**

Maternal satisfaction after a caesarean section (CS) is a key quality indicator, yet understudied in low-income and middle-income countries. This study used a qualitative method approach to address that gap, by exploring factors influencing maternal satisfaction in South African government hospitals, focusing on pain management, emotional support and communication, within the local context of KwaZulu-Natal.

**Methods:**

A qualitative descriptive study design was employed. Women who underwent CS in two government hospitals in Durban were included in the study. Data were collected using questionnaires for socio-demographic information and semi-structured interviews. Thematic analysis was used to identify and code themes related to maternal satisfaction.

**Results:**

A total of 100 women were interviewed. Three-quarters of the women (*n* = 77) were satisfied with their CS experience, with only one participant expressing dissatisfaction. Maternal satisfaction was likely primarily related to positive delivery outcomes. Factors that improved satisfaction included effective pain management, a supportive ward atmosphere, and interactions with peers. Despite overall positive responses, only one-third were willing to undergo CS in the future. Maternal satisfaction was reduced by issues like insufficient preoperative information, language barriers, limited autonomy, and a lack of breastfeeding or emotional support.

**Conclusion:**

Most women reported satisfaction with their CS experience, yet expressed reluctance to undergo the procedure again. This paradoxical phenomenon highlights the complexity and multifaceted nature of maternal satisfaction. Healthcare providers should prioritise open communication, empathy, and respect for women’s preferences and values to provide high-quality, patient-centred care.

**Contribution:**

The study highlighted the need for responsive, equitable, and person-centred approaches to CS care, particularly within resource-constrained public health settings. By addressing the identified gaps and implementing targeted, theory-informed strategies, healthcare providers and policymakers can take meaningful steps towards improving maternal experiences and outcomes across South Africa’s government hospitals.

## Introduction

Maternal satisfaction following caesarean section (CS) is a key indicator of healthcare quality, particularly in resource-limited settings such as South Africa, where systemic challenges can intensify negative maternal experiences.^1^ Maternal satisfaction following CS is an important patient-centred indicator of healthcare quality and should be interpreted alongside other measures such as clinical outcomes, safety, accessibility, and equity to provide a comprehensive assessment of care quality. Despite its significance, maternal satisfaction with CS remains underexplored in low- and middle-income countries (LMICs), with limited local data highlighting deficiencies in emotional and physical support, communication and pain management within public hospitals.^2^ This is concerning, as negative CS experiences are associated with poor emotional well-being, increased risk of postpartum depression and impaired mother–infant bonding.^3,4^

Inadequate preoperative counselling, poor communication and insufficient emotional reassurance heighten maternal anxiety and dissatisfaction, which aligns with literature on the psychosocial aspects of childbirth.^5,6^ Gaps in postpartum care, especially in breastfeeding support and personalised counselling, highlight how healthcare structures and process-related healthcare limitations affect maternal satisfaction, as illustrated by Donabedian’s model.^7^ There is a need for supportive environments that help mothers make sense of and cope with the childbirth experience. Comprehensive care should address not just physical, but also emotional and social needs.^8^ Similarly, the biopsychosocial model shows that maternal well-being depends on a blend of biological, psychological and social support, including adequate pain relief, emotional reassurance and family involvement.^9^ When any of these elements are lacking, the mother’s recovery and satisfaction can be adversely affected.

By describing these deficits through patient-centred care that prioritises effective communication, emotional support and comprehensive postoperative care, this study will contribute to improvements in maternal healthcare quality in LMICs and align with global efforts to enhance maternal outcomes and satisfaction.^10^ This study aims to address this research gap by evaluating maternal emotional experiences before, during and after CS. It focuses on assessing communication quality, perceived autonomy in decision-making, efficacy of pain management strategies and the quality of postoperative care, including breastfeeding support and self-care guidance.

## Research methods and design

### Research philosophy

This study is grounded in interpretivism, a research philosophy founded on the understanding of human phenomena through exploring subjective meanings, values and beliefs individuals place within their social settings.^11^ This was also conducive to in-depth examination of how women perceived their sense of control, communication with healthcare providers and emotional well-being. This was also compatible with the biopsychosocial model, which stresses emotional, psychological and social dimensions of maternal care.

### Study design

We employed a qualitative design, which explores subjective meanings and personal experiences, ideal for studying complex events such as childbirth, where individual perceptions and emotional reactions are crucial. This structure harmonised with the interpretivist orientation of the study, permitting participants’ narratives to direct meaning without forcing prior theoretical frameworks.

### Target population and setting

The study population comprised women who had undergone CS at Prince Mshiyeni Memorial Hospital and Mahatma Gandhi Memorial Hospital in the eThekwini district in KwaZulu-Natal. Women < 18 years of age and those who had vaginal deliveries were excluded.

Purposive sampling was used to recruit 100 women ≥ 18 years old, on their second day post-CS in the postnatal ward. The decision to recruit 100 participants was informed by the need to achieve data saturation, a key principle in qualitative research that refers to the point at which no new themes or insights emerge from the data. Given the relatively high frequency of CS deliveries in the participating hospitals, we hoped to capture diverse and meaningful responses.

This method enabled the collection of rich, context-specific data, supporting the study’s aim to explore maternal experiences and perceptions immediately following CS. Participants were recruited with the assistance of the postnatal ward nurses following a briefing on the study’s objective and eligibility criteria.

### Data collection

A semi-structured interview schedule was employed, and responses were recorded using a structured instrument that included socio-demographic questions and a semi-structured interview guide for preoperative, intraoperative and postoperative experiences. The instrument underwent pretesting that involved consulting 10 senior clinicians in the anaesthetic department to ensure the questionnaire was both valid and comprehensible. Their input was not intended to alter the study objectives but rather to strengthen the methodological rigour of the data collection tool. Specifically, they reviewed the wording of questions to confirm that items were phrased clearly, avoided ambiguity, and accurately reflected the intended constructs. We further administered the questionnaire to 10 women post-CS to assess their understanding of the questions. The feedback obtained facilitated modifications to enhance the clarity and inclusivity of first-time mothers. These amendments enhanced the instrument’s appropriateness and reliability of the instrument. Face-to-face interviews were conducted with the principal researcher, with each interview lasting between 15 min and 30 min. Interviews were conducted in a private room to maintain confidentiality and minimise the impact of external factors on the responses of the participants. Recruitment occurred from 04 March to 17 March 2024.

The semi-structured interview questions were administered in English, which was the language of the research instrument. The researcher was fluent in both English and isiZulu and was able to facilitate communication and provide clarification when necessary. Despite the linguistic diversity, most participants expressed comfort with English during the interviews, and no significant barriers to comprehension were reported.

### Data analysis process

Thematic analysis was used to systematically analyse the data, facilitating the identification and interpretation of meaningful themes and patterns.^12^ This method is best applied in descriptive qualitative research, allowing for comparison of experiences while preserving individual depth and context. The analysis involved data familiarisation, generation of codes, theme development and interpretation.

### Trustworthiness of the study

The study adhered to the Lincoln and Guba standards of trustworthiness^13^: credibility, transferability, dependability, and confirmability, with additional guidance from Haq Rasheed’s contemporary perspective.^14^ Credibility strategies included extended engagement, data triangulation through interviews and literature, and member-checking to validate interpretations.^15,16^ Confirmability was maintained through reflexivity and the use of direct quotations to ground findings in lived experience.^14,17^

### Ethical considerations

The study was conducted in adherence to the protocol, the principles of the Declaration of Helsinki, the International Conference on Harmonisation’s Good Clinical Practice guidelines and all applicable South African laws and regulations. Ethical approval was obtained from the University of KwaZulu-Natal Biomedical Research Ethics Committee (BREC/00005734/2023), the relevant hospitals and the Department of Health Ethics Review Committee. There was no physical risk in the study; however, discussing childbirth experiences could potentially cause emotional distress. In such cases, participants were offered referrals to psychologists for additional support. The findings of this qualitative study were reported in compliance with the Standards for Reporting Qualitative Research recommendations.^18^

## Results

A total of 100 participants were interviewed; 60 participants from Prince Mshiyeni Memorial Hospital and 40 participants from Mahatma Gandhi Memorial Hospital. The median (interquartile range) age was 28 years (18–43 years) and median parity was 2 (1–7). None of the participants reported a history of depression or anxiety (Table 1).

**TABLE 1:** Participants’ sociodemographic characteristics (*N* = 100).

Characteristics	Variable	*n*	%
Education	Primary education	1	1
	Secondary education	87	87
	Higher education	12	12
Employment	Scholar or Student	8	8
	Unemployed	66	66
	Employed	26	26
Marital status	Unmarried	95	95
	Engaged	3	3
	Married	2	2
Known depression and/or anxiety	Yes	0	0
	No	100	100

Previous exposure to anaesthesia was included as a participant characteristic to capture its potential influence on maternal perceptions and experiences. Prior exposure to anaesthesia can shape expectations and reduce anxiety, as women familiar with the process may feel reassured and more in control. Conversely, those without such exposure may experience heightened fear and uncertainty. This variable was used descriptively to contextualise participant experiences rather than to define the study setting, thereby strengthening the interpretation of findings related to psychological preparedness and patient-centred care.

**FIGURE 1:**
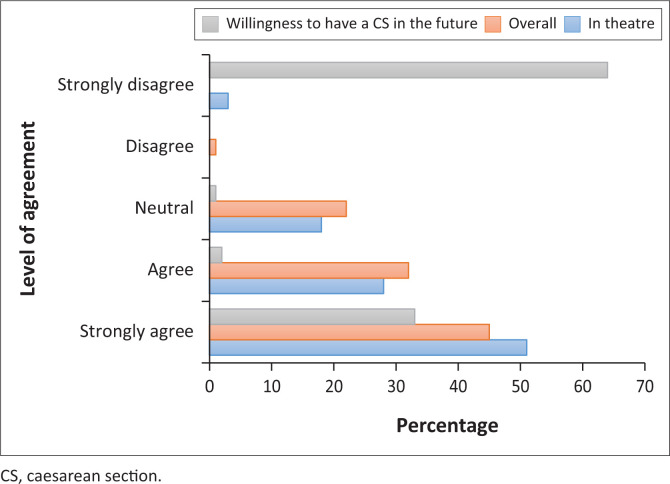
Participants’ satisfaction and willingness to have a future caesarean section.

Figure 1 shows levels of participant satisfaction with in-theatre experience, overall care, and willingness to have a future CS. Most participants were satisfied in-theatre 79% (51% strongly agree and 28% agree), with overall satisfaction found in 77% (45% strongly agree and 32% agree). However, most participants were strongly unwilling to have a CS in future (64% strongly disagree). Contrary to this, the 35.00% of participants expressed willingness to undergo CS again (33% strongly agreeing and 2% agreeing). Neutral and disagree responses demonstrate the spectrum of people’s experiences and expectations.

The term ‘in-theatre’ refers specifically to participants’ experiences during the CS procedure itself, within the operating theatre environment. It encompasses their perceptions of intraoperative care, including pain management, communication with staff, and feelings of safety while undergoing surgery.

Overall satisfaction refers to the woman’s holistic appraisal of her CS experience across the entire perioperative continuum (preoperative, intraoperative, and postoperative) and encompasses both clinical and non-clinical dimensions of care.

Satisfaction with the in-theatre experience was reported to be high among participants (79%), with several key factors contributing to this positive perception. Effective pain management emerged as a central component, as mothers consistently found that intraoperative pain was well controlled through appropriate anaesthetic techniques. The absence of pain during surgery fostered a sense of reassurance and confidence, reinforcing trust in the clinical team. In addition to pain control, communication within the theatre environment played a significant role in shaping maternal satisfaction.

Participants valued clear explanations, reassurance from staff, and respectful interactions, which collectively reduced anxiety and enhanced their sense of involvement in the process. Furthermore, feelings of safety were repeatedly emphasised, with mothers describing the theatre as a secure environment where they were closely monitored and supported. This combination of physical comfort, effective communication, and psychological reassurance underscores the multidimensional nature of theatre satisfaction, highlighting that maternal experiences are shaped not only by clinical outcomes but also by interpersonal dynamics and the broader atmosphere of care. The participants previous and current delivery characteristics are summarised in Table 2.

**TABLE 2:** Participants’ previous and current delivery characteristics (*N* = 100).

Characteristics	*n*	%
**Previous mode of delivery**		
Not applicable	31	31
Natural vaginal delivery	28	28
Caesarean section	32	32
Both	9	9
**Previous anaesthetic exposure**		
Yes	42	42
No	58	58
**Previous anaesthetic technique**		
Spinal anaesthesia	41	41
General anaesthesia	1	1
Both spinal and general anaesthesia	1	1
Not applicable	57	57
**Urgency of the current surgery**		
Elective	31	31
Emergency	69	69
**Current anaesthetic technique**		
Spinal anaesthesia	98	98
General anaesthesia	2	2

### Thematic data analysis findings

Thematic data analysis generated five key themes with codes from the collected interview data (Table 3).

**TABLE 3:** Themes and codes generated from the interview data.

Theme description	Codes
Emotional experiences surrounding caesarean section	Anxiety and indifference before the procedure Emotional relief and joy after delivery Postoperative concerns about baby separation and scarring
Quality and comprehensiveness of communication	Limited or unclear preoperative information about caesarean section, risks and anaesthesia Lack of active involvement in discussions or decision-making Minimal updates during the procedure
Perceptions of control and decision-making	Lack of control and involvement in decisions Compliance with instructions without room for preferences or questions
Pain management	Effective intraoperative and postoperative pain control via injections Satisfaction with pain management’s role in recovery and mobility
Postoperative care and support	Limited breastfeeding support Satisfactory ward cleanliness and food, though dietary needs are not addressed Peer support among mothers fostering shared recovery

#### Theme 1: Emotional experiences surrounding caesarean section

Participants expressed diverse emotional responses before, during and after the CS, including fear, anxiety, indifference or relief. Preoperatively, many expressed apprehension and uncertainty. One participant stated:

‘I was scared because I did not know what to expect.’ (P3, 18 year old, scholar)

While another shared:

‘I wanted to cry. I was so scared because I did know what to expect.’ (P46, 24 year old, unemployed)

These responses underscore the need for enhanced communication and reassurance to reduce preoperative anxiety.

Intraoperatively, many participants reported feelings of lack of control and detachment, with one stating:

‘You just do what you are told to do.’ (P1, 18 year old, unemployed)

Postoperatively, emotions were predominantly positive when the baby was delivered safely. One participant opined:

‘I heard baby crying and I was so happy. I knew everything was okay because if the baby doesn’t cry it means there is a problem.’ (P67, 39 year old, cashier)

Another echoed this:

‘I was happy that I have delivered and relieved that my baby is alive.’ (P62, 33 year old, unemployed)

This underscores the emotional significance of positive delivery outcomes. However, not all experiences were positive. Some participants experienced distress because of neonatal complications or immediate separation from their newborns. One participant described:

‘I was very sad … Baby came out not crying and had breathing problems was taken to nursery.’ (P90, 40 year old, unemployed)

#### Theme 2: Quality and comprehensiveness of communication

Participants frequently reported inadequate communication regarding the procedure, risks and their preferences. One participant explained:

‘I was told about the injection that it will make my legs numb. No other information … I was not given preferences.’ (P14, 32 year old, admin clerk)

Language barriers further complicated understanding:

‘I did not understand they spoke English and no one to translate for me.’ (P51, 29 year old, unemployed)

Several participants felt that explanations were rushed or incomplete. One of the participants stated:

‘No. They did not make time to explain adequately to me.’ (P38, 28 year old, unemployed)

This lack of information left patients feeling unprepared and anxious. However, a few participants reported more positive experiences:

‘Yes. The information was clear … I had no problem because I knew what to expect.’ (P7, 36 year old, unemployed)

Another participant added:

‘Yes. They explained everything very well.’ (P16, 31 year old, unemployed)

The indications for CS in this study were diverse, with nearly half (48%) performed for foetal distress. Other indications included hypertensive disorders such as eclampsia (2%) and pre-eclampsia (5%), previous CS with varying clinical considerations (totalling approximately 27%), macrosomia (5%), twin pregnancy (4%), breech presentation (3%), and a small proportion of cases because of poor progress, cord presentation, cephalopelvic disproportion, antepartum haemorrhage, preterm labour, and failed induction. Women undergoing emergency CS often perceive limited autonomy, as decisions are clinician-driven and urgent, whereas those with elective indications may experience greater involvement in decision-making.

#### Theme 3: Perceptions of control and decision-making

A common theme among participants was the perceived lack of autonomy and involvement in decision-making. One participant stated:

‘No. I was not given a chance to say what I prefer.’ (P1, 18 years, unemployed)

Another noted:

‘No I did not have an ability to express my preferences. I was told that I will have injection at the back … No. I did not feel sense of control.’ (P2, 18 year old, scholar)

One participant stated:

‘The doctor told me to sit so that he can inject me on my spine … I did not feel sense of control. I was told what to do. There was no discussion.’ (P30, 38 year old, unemployed)

Similarly, another participant shared:

‘I was in pain and I had to lie still in bed for 6 hours without moving.’ (P8, 24 year old, unemployed)

The accounts provided by participants underscore a profound sense of disempowerment arising from the lack of autonomy and involvement in decision-making during their CS experiences. Several mothers described being instructed on what to do without any opportunity to express preferences or engage in dialogue with healthcare providers. For example, participants reported being told to adopt specific positions for spinal injections or to remain immobile for extended periods, with no discussion of alternatives or consideration of their comfort. Such experiences highlight a paternalistic approach to care, where clinical directives overshadow patient agency. This absence of shared decision-making not only diminished women’s sense of control but also negatively influenced their overall satisfaction, reinforcing the importance of fostering participatory care models that respect maternal voice and choice in clinical settings.

However, a minority of participants reported more positive experiences, citing clear explanations:

‘Yes. They explained everything very well… I was told that my legs will be numb. I was not told about the complications of spinal anaesthesia.’ (P16, 31 year old, unemployed)

Other participants shared:

‘Yes. They answered lot of my questions.’ (P92, 25 year old, unemployed)

‘Yes. When I ask questions, I was given answer.’ (P94, 32 year old, unemployed)

#### Theme 4: Pain management

Most participants expressed satisfaction with both intraoperative and postoperative pain management. One participant highlighted the effectiveness of pain management stating:

‘I had pain on day 1. I was given injection.’ (P99, 38 year old, unemployed)

Another echoed this sentiment:

‘I was in severe pain. I was given injection and tablets.’ (P79, 32 year old, unemployed)

However, some participants highlighted gaps in communication regarding pain management and anaesthesia risks. One participant shared:

‘No I was not told about the risk of CS … I was only told that I must sit upright and bend my head and not move. They did not tell about the risk and complications of spinal anaesthesia.’ (P12, 37 year old, human resources officer)

A few participants reported inadequate or delayed pain relief. One participant stated:

‘I was given injection on my arm. Yes. I had no pain for 6 hours. The pain came back again and I was given tablets.’ (P5, 28 year old, unemployed)

Another participant opined:

‘No, spinal injection did not work.’ (P63, 27 year old, unemployed)

#### Theme 5: Postoperative care and support

Participants’ experiences of postoperative care varied. Some participants praised the support received:

‘Yes there is assistance available, and it is excellent. The sisters are helpful.’ (P2, 18 year old, scholar)

Another echoed this, saying:

‘I would say it is good. The assistance is readily available.’ (P1, 18 year old, unemployed)

‘No. I did not receive breastfeeding support. I wish they can give more information about breastfeeding.’ (P2, 18 year old, scholar)

While another added:

‘No. I did not get breastfeeding support or information about breastfeeding.’ (P3, 18 year old, scholar)

Participants also found disparities in wound care education and self-care. One participant shared that she was advised that she:

‘[*S*]houldn’t bend when walking so that the wound will heal faster.’ (P53, 29 year old, unemployed)

The statement reflects the participant’s personal account of advice received during postoperative care, rather than evidence-based guidance. The rationale for such advice is unclear and may contradict established recommendations, highlighting the need for further investigation into the consistency and evidence base of postoperative instructions provided to mothers.

Another participant also commented they got:

‘Tips about wound care.’ (P56, 18 year old, unemployed)

One participant stated:

‘My family visits and calls to check us. Baby daddy is also supportive as well.’ (P4, 22 year old, unemployed)

Peer interactions within the ward environment were highly valued and provided emotional support, with one stating:

‘We help each other if someone is still have pain. We borrow each other things like phone charger.’ (P87, 28 year old, college student)

## Discussion

The study’s demographic findings highlight key factors influencing maternal satisfaction with CS in South African government hospitals. Most participants were young, had secondary-level education and were unemployed (66%), reflecting vulnerabilities related to limited childbirth experience, health literacy and socioeconomic challenges. Most women (69%) underwent emergency CS, which is often linked to heightened anxiety and less preparation, and the majority (98%) received spinal anaesthesia, a preferred method associated with effective pain management. None reported pre-existing mental health conditions, which provided a clear baseline for evaluating care. While the majority underwent emergency CS, which is often linked to heightened anxiety and less preparation, nearly all received spinal anaesthesia, a preferred method associated with effective pain management.

An important feature of the study sample was the high proportion of women who reported being single at the time of delivery. This demographic profile differs from the broader population norms often reported in maternal health literature, where marital or cohabiting partnerships are more frequently represented. The predominance of single mothers in this study is noteworthy, as it may influence both the experiences and expectations of CS care.

Unmarried women may face unique challenges, such as reduced access to consistent emotional or financial support, which are critical factors influencing maternal well-being and satisfaction.^2^ Marital status can also shape expectations of care, as women with supportive partners may anticipate greater inclusion in decision-making processes and higher levels of communication.^5^ This implies that marital status may have a potential influence on maternal satisfaction and emotional experiences during CS.

This prospective, qualitative study showed that while most women are satisfied with their overall experience of CS (77% [45% strongly agree and 32% agree]), almost two-thirds feel strongly that they would not undergo the procedure again. This apparent contradiction reflects the complex interplay between clinical outcomes, patient expectations, and long-term recovery experiences. Satisfaction was often linked to effective anaesthetic management, supportive theatre staff, and the perception of safety during the procedure. However, negative recollections of postoperative pain, delayed recovery, and inconsistent communication with healthcare providers shaped future preferences, leading some mothers to reject CS as a desirable mode of delivery despite acknowledging positive aspects of their care. This paradox underscores the distinction between situational satisfaction derived from immediate experiences of safety and professionalism and retrospective evaluation, which incorporates the broader physical and emotional consequences of surgery. It highlights the need for healthcare systems to address not only intraoperative care but also postoperative pain management, emotional support, and communication, thereby aligning short-term satisfaction with long-term maternal preferences and well-being.

The participants in the study experienced a range of emotions before, during and after the CS. These emotions included fear, anxiety, indifference, and relief. Before surgery, many felt apprehensive and uncertain about what to expect. A study confirmed that women who underwent emergency CS experienced a range of psychosocial outcomes, including post-traumatic stress, anxiety and depression.^19^ Emergency CS is generally associated with anxiety, inadequate preparation and limited maternal agency.^2,6^ Elective CS, in contrast, involved pre-counselling, fully informed decision-making and increased emotional preparation, with higher satisfaction.^5^

Our study highlighted issues with the quality and comprehensiveness of communication regarding CS, risk, and their preferences. Many felt that explanations were inadequate, rushed, or incomplete, leading to feelings of being unprepared and anxious. Language barriers further complicated understanding for some participants. However, a few participants reported positive experiences, with clear communication. Effective communication is crucial for patient satisfaction and reducing anxiety in healthcare settings.^20^ The study emphasises that clear communication can improve patient outcomes and experiences.

Mothers should receive clear, timely explanations about the indication for CS, the anaesthetic plan, and the sequence of events in theatre.

Communication is not limited to clinical facts; it also involves empathetic reassurance. Simple gestures such as checking on comfort, acknowledging fears, and offering encouragement contribute significantly to maternal confidence and satisfaction.

Even in emergency contexts, where time is limited, communication should include efforts to involve mothers in decisions to the extent possible. This reinforces autonomy and mitigates feelings of helplessness.

Communication should extend beyond the theatre into the postoperative period, ensuring mothers receive consistent guidance on pain management, wound care, and breastfeeding support. The findings of this study highlight gaps in these processes, particularly in emergency situations, underscoring the need for structured communication protocols.

The perceptions of control and decision-making reveal participants’ feelings of a lack of autonomy and involvement in the decision-making processes regarding their care. Participants illustrate this lack of control, where they were not given opportunities to express preferences or were told what to do without discussion. This experience of disconnection is linked to reduced patient engagement. Existing evidence supports the importance of patient involvement in care decisions for better outcomes. A study by Epstein and Street^20^ emphasises the role of effective clinician-patient communication in fostering patient engagement and better health outcomes.

Pain management is a critical aspect of perioperative care. Most participants expressed satisfaction with both intraoperative and postoperative pain management, citing the effectiveness of pain relief methods such as injections and tablets in managing pain and positively impacting mobility and recovery. However, some participants noted gaps in communication regarding pain management and anaesthesia risk. Some women reported inadequate pain relief after returning from theatre, experiencing unacceptable levels of pain before receiving their next scheduled pain medication. Evidence that supports the importance of effective pain management suggests that inadequate pain relief can lead to decreased mobility and recovery.^21^ Another study emphasises the role of the placebo effect in pain management, suggesting that patient expectations and conditioning can influence pain perception.^22^ Postoperative pain is generally poorly assessed, and treatment is often poorly administered in South African hospitals.^23^

Postoperative care experiences varied among participants, with some praising the support received from the healthcare staff, particularly noting the helpfulness of the nurses. However, disparities in breastfeeding support and wound care education were highlighted, with some participants expressing a desire for more information on breastfeeding and specific guidance on wound care to aid in faster healing. Studies emphasise that effective pain management and proper wound care can reduce complications and promote healing.^24^

In addition, breastfeeding support is crucial for both maternal and infant health, with evidence showing that breastfeeding assistance can improve breastfeeding rates and reduce postpartum complications.^25^ The qualitative findings of this study highlighted gaps in breastfeeding support among post-CS mothers, particularly in the immediate postoperative period when pain, immobility, and surgical recovery can hinder initiation. While these accounts suggest that, although patients face unique challenges requiring tailored assistance, the study design does not allow for definitive conclusions about whether inadequate breastfeeding support is a problem exclusive to post-CS women or a broader issue affecting all post-delivery mothers. Addressing this question would require a quantitative study comparing breastfeeding support across delivery modes. Nonetheless, the qualitative evidence presented here underscores the importance of ensuring universal access to breastfeeding support, while recognising that post-CS women may require additional, structured interventions to overcome specific barriers. Peer interactions and family support also play a significant role in emotional support during the postoperative period, contributing to better maternal outcomes.

Despite reporting high levels of overall satisfaction with their CS, a significant proportion of participants expressed reluctance to undergo a repeat CS in the future. Participants who reported resistance to CS referred to unpleasant experiences in terms of pain, fear or emotional distress. This finding is supported by other studies in which women who expressed reluctance to undergo a repeat CS frequently attributed their resistance to unpleasant experiences.^2,3^ In this study, interviews were conducted on day 2 post-operation, a period when mothers were still in the early stages of recovery. It is possible that some participants were still experiencing pain, fatigue, or emotional distress, which may have shaped their perceptions of CS as an unpleasant experience. This context is acknowledged as a limitation because maternal views may evolve over time as recovery progresses and as mothers gain perspective on their birth experience.

However, capturing perceptions at this early stage was intentional, as it allowed the study to document immediate postoperative experiences and anxieties, which are critical to understanding maternal satisfaction and the need for supportive interventions. Longer-term follow-up could indeed reveal shifts in perception. Future research incorporating longitudinal designs would therefore be valuable to explore how maternal satisfaction and perceptions of control change from the immediate postoperative period to later stages of recovery.

Participants who expressed willingness to undergo a repeat CS credited their positive experience to effective pain management, previous positive CS experiences, emotional support and interpersonal care. These findings are consistent with existing studies.^5^ Neutral comments expressed ambivalence or unresolved experiences. These diverse responses call for understanding and addressing drivers of dissatisfaction, with encouragement of positive factors.

Our findings indicate that maternal satisfaction with CS care is not so much an articulation of a clinical outcome, but more a result of multidimensional processes between individual characteristics, communication processes, healthcare organisations and emotional processes. Ffrench-O’Carroll et al. demonstrated that while recovery scores correlated with satisfaction, interpersonal communication and reassurance were stronger determinants of maternal perceptions of care.^26^ Similarly, Jikijela et al. highlighted that maternal satisfaction in South African public hospitals was shaped by emotional support, midwife interactions, and organisational factors, rather than clinical outcomes alone.^27^ These findings reinforce the plausibility of this study’s interpretation, situating maternal satisfaction within a broader biopsychosocial framework.

### Limitations of the study

The study was conducted at only two government hospitals, which may limit the generalisability of the findings to other healthcare settings. Furthermore, the semi-structured interviews were conducted while participants were still hospitalised, which may have influenced their responses compared to interviews conducted post-discharge in a home environment. This could have affected participants’ reflections on their experiences, potentially introducing response bias.

### Implications or recommendations

Enhancing maternal satisfaction with South African CS care necessitates clear, literacy-sensitive communication, shared decision-making and ongoing emotional support. Standardised postoperative care, such as breastfeeding advice and informed pain management, is necessary. Utilising holistic care models will close systemic loopholes and improve patient-centred outcomes in public hospitals. We recommend the following be considered:

- Healthcare providers should use simple, clear language and provide written information in multiple languages to ensure that all patients understand their care. Ensure that translation services are available to facilitate communication. This must also be provided in antenatal clinics.
- Healthcare providers should involve patients early in decision-making processes, considering their preferences, values and beliefs. Ensuring that healthcare providers offer clear, timely, and comprehensible explanations of the medical rationale for CS, while also acknowledging maternal preferences and concerns. Incorporating structured counselling sessions, decision aids, and opportunities for dialogue can help bridge the gap between clinical necessity and maternal agency.
- Offer emotional support and counselling to patients throughout their care journey.
- Implement standardised postoperative care protocols, including pain management and breastfeeding advice.
- Implement regular exit surveys and feedback mechanisms to assess patient satisfaction and identify areas for improvement.
- Identify and address systemic gaps, such as inadequate staffing, insufficient resources, and inefficient processes, to improve patient-centred outcomes.
- Integrate breastfeeding counselling and support services into routine maternity care for all mothers, regardless of delivery mode.
- Provide specialised assistance for post-CS mothers, including bedside support during the immediate postoperative period.
- Train healthcare providers to recognise the unique challenges faced by mothers in the postoperative period and to offer tailored interventions that promote successful breastfeeding.
- Ensure continuity of breastfeeding support from theatre recovery through discharge, with follow-up services available in community clinics. Embed breastfeeding support into standardised postoperative care protocols, thereby reducing disparities and improving both maternal satisfaction and neonatal outcomes.

## Conclusion

Most women reported satisfaction with their CS experience, yet expressed reluctance to undergo the procedure again. This paradoxical phenomenon highlights the complexity and multifaceted nature of maternal satisfaction. Healthcare providers should prioritise open communication, empathy, and respect for women’s preferences and values to provide high-quality, patient-centred care. Maternal satisfaction is a required obstetric care quality metric with implications for health outcomes and institutional trust. Implementing these theory-driven, patient-centred interventions in resource-poor environments is required for improving CS experiences and maternal healthcare in South African government hospitals.
